# Voice analysis and deep learning for detecting mental disorders in pregnant women: a cross-sectional study

**DOI:** 10.1007/s44192-025-00138-0

**Published:** 2025-02-08

**Authors:** Hikaru Ooba, Jota Maki, Hisashi Masuyama

**Affiliations:** Department of Obstetrics and Gynecology, Okayama University Graduate School of Medicine, Dentistry and Pharmaceutical Sciences, 2-5-1 Shikata-cho, Kita-ku, Okayama, Okayama 700-8558 Japan

**Keywords:** Perinatal mental disorders, Voice analysis, Machine learning, Screening, Pregnant women

## Abstract

**Introduction:**

Perinatal mental disorders are prevalent, affecting 10–20% of pregnant women, and can negatively impact both maternal and neonatal outcomes. Traditional screening tools, such as the Edinburgh Postnatal Depression Scale (EPDS), present limitations due to subjectivity and time constraints in clinical settings. Recent advances in voice analysis and machine learning have shown potential for providing more objective screening methods. This study aimed to develop a deep learning model that analyzes the voices of pregnant women to screen for mental disorders, thereby offering an alternative to the traditional tools.

**Methods:**

A cross-sectional study was conducted among 204 pregnant women, from whom voice samples were collected during their one-month postpartum checkup. The audio data were preprocessed into 5000 ms intervals, converted into mel-spectrograms, and augmented using TrivialAugment and context-rich minority oversampling. The EfficientFormer V2-L model, pretrained on ImageNet, was employed with transfer learning for classification. The hyperparameters were optimized using Optuna, and an ensemble learning approach was used for the final predictions. The model’s performance was compared to that of the EPDS in terms of sensitivity, specificity, and other diagnostic metrics.

**Results:**

Of the 172 participants analyzed (149 without mental disorders and 23 with mental disorders), the voice-based model demonstrated a sensitivity of 1.00 and a recall of 0.82, outperforming the EPDS in these areas. However, the EPDS exhibited higher specificity (0.97) and precision (0.84). No significant difference was observed in the area under the receiver operating characteristic curve between the two methods (p = 0.759).

**Discussion:**

The voice-based model showed higher sensitivity and recall, suggesting that it may be more effective in identifying at-risk individuals than the EPDS. Machine learning and voice analysis are promising objective screening methods for mental disorders during pregnancy, potentially improving early detection.

**Conclusion:**

We developed a lightweight machine learning model to analyze pregnant women's voices for screening various mental disorders, achieving high sensitivity and demonstrating the potential of voice analysis as an effective and objective tool in perinatal mental health care.

## Introduction

Mental disorders during pregnancy are a significant public health concern, affecting approximately 10–20% of pregnant women worldwide [1]. Conditions such as depression, anxiety, and bipolar disorder can have detrimental effects on both maternal well-being and obstetric outcomes, including preterm birth, low birth weight, and complications during delivery [2, 3]. Early detection and intervention are essential to mitigate these risks and improve health outcomes for both the mother and child. Traditional screening tools, such as the Edinburgh Postnatal Depression Scale (EPDS) [4] and Brief Symptom Inventory 18 (BSI18) [5], rely on self-reported questionnaires. Although useful, these methods have limitations, including subjectivity, potential for social desirability bias, and dependence on the patient's ability and willingness to accurately report symptoms [6]. Additionally, healthcare providers may encounter challenges in administering these assessments owing to time constraints and a lack of familiarity with the tools [7]. Consequently, there is an urgent need for objective, efficient, and easily deployable screening methods for perinatal care.

Research has demonstrated correlations between vocal attributes and mental health conditions, with studies showing that changes in speech patterns, such as reduced pitch variability, slower speech rates, and altered energy levels, can indicate depression and anxiety. Research has highlighted the correlation between vocal attributes and mental disorders. Studies indicate a 32% prevalence of depression, anxiety, and somatic concerns in patients with voice problems [8], with depressive symptoms doubling the probability of voice issues [9]. In younger women, voice disorders such as spasmodic dysphonia (SD), functional dysphonia (FD), and psychogenic dysphonia (PD) are more prevalent [10]. SD has a prevalence of 3.5–7.0 per 100,000, with women at higher risk, particularly around the average onset age of 30 years, coinciding with childbearing years [11]. Approximately 42% of patients with SD have comorbid mental disorders [12], and the risk of depression or anxiety disorders is similar between patients with SD and those with other voice disorders [13]. These findings suggest the potential of voice as a valuable tool for screening mental disorders in pregnant women. Machine learning algorithms can identify subtle vocal changes, offering a promising avenue for objective mental health screening [14].

However, existing research has predominantly focused on the general population and specific disorders, such as depression, with limited attention to pregnant women and a broader range of mental disorders [15]. Moreover, challenges such as data imbalance in minority classes (e.g., specific mental disorders among pregnant women) can compromise the performance of machine learning models if not properly addressed [16]. Simple accuracy metrics may be misleading in imbalanced datasets, highlighting the importance of using balanced performance metrics to evaluate model efficacy. The objective of this study was to develop a lightweight machine learning model that analyzes the voices of pregnant women to screen for various mental disorders, thereby addressing the limitations of current screening tools. Given the potential application of this model in real-time screening, such as on edge devices with limited computational resources, it is essential to prioritize both accuracy and efficiency. By adopting a balanced data approach and focusing on this specific population, we aimed to create an objective, efficient, and specialized tool that can enhance perinatal mental health screening practices.

## Material and methods

### Study design and participants

We conducted a cross-sectional study at a single center in Japan. The study population comprised pregnant women who delivered at our hospital between August 2022 and April 2023 and attended a one-month postnatal checkup. In Japan, all postpartum women are recommended to receive a one-month postpartum checkup. Eligible participants were those who had been pregnant after the 13th week of gestation. Exclusion criteria included miscarriage before the 12th week of pregnancy, refusal to consent to audio recording, incomplete audio recording, or missing data. Based on previous studies that estimated a 14% comorbidity rate of mental disorders during pregnancy [2], we aimed to recruit 25 pregnant women with mental disorders. Considering a dropout margin of 10%, the total sample size was set at 200 participants.

### Settings for voice recording

Voice samples were collected in a natural consultation room setting during the one-month post-delivery checkup. We used a SONY PCM D-100™ recorder, which records at 24-bit linear PCM quality with a 22.05 kHz sampling rate in uncompressed WAV format. The recorder was placed discreetly outside of the participants' views to ensure a natural environment. There were no limitations on the duration or subject matter of the recorded conversations. To capture comprehensive interactions, we recorded not only the voices of the pregnant women but also those of clinicians, infants, and third parties, as well as ambient sounds.

### Collection of background information

Sociomedical data and EPDS scores were obtained from participants' medical records. Before the study, psychiatric disorders were confirmed through diagnoses made by independent experts. Participants were categorized into Class 1 if diagnosed with any psychiatric disorder prior to the current childbirth and into Class 0 if no such diagnosis was present. Of these participants with a psychiatric disorder, diagnoses had been made prior to or during pregnancy by board-certified psychiatrists in accordance with DSM-5 criteria; however, medical records did not specify the precise timing or type of structured interview for each case. Consequently, the exact timing of the diagnostic interviews varied among participants, and no additional formal assessment was conducted for those who did not carry a previously identified psychiatric diagnosis. For this study, we therefore relied on the presence or absence of a psychiatric disorder documented before delivery as the label for each participant.

### Noise reduction

Audio data that were not successfully recorded were excluded from the analysis. We used the Demucs model [17], known for its audio source separation capabilities, to filter out specific auditory elements, such as human voices, from background noise. Audition CC 2022® was used to remove voice signals that were not from the primary subjects, such as doctors or companions. Editing tasks were carefully performed manually. The data were randomly stratified, allocating 60% for training, 15% for validation, and 25% for testing.

## Theory/calculation

### Audio segmentation

Conversational audio, characterized by a variety of elements such as laughter and tension indicators, poses challenges for accurate class prediction due to its complexity. To address this, we segmented the audio data into smaller units. Each voice sample was divided into 5,000 ms intervals, with predictions made for each segment. These individual segment predictions were then integrated to determine the overall class for the entire conversation [18]. To diversify our training data, we introduced a 'shift' operation, randomly altering the start and end points of each segment within a 0–2,500 ms range, creating overlapping segments with the original data. For training purposes, we augmented the data by producing both the original segment and four shifted variants. This shift operation was employed only for the training dataset.

### Conversion to melspectrograms

We conducted a short-time Fourier transform (STFT) [19] on all segments to produce spectrograms. To mitigate the trade-off between the time and frequency resolutions inherent in spectrograms [20], we generated three distinct spectrograms using window widths of 512, 1,024, and 2,048, each with corresponding overlaps. Given that human auditory perception is more sensitive to lower frequencies [21], we created mel spectrograms by applying an mel filter bank with 128 filters to the original spectrograms. These mel spectrograms were converted into decibel intensities, normalized to a range between –1 and 1, and resized to 224 × 224 pixels. The mel spectrograms were combined to form a three-channel spectrogram image aligned with the perceptual characteristics of human hearing.

### Data augmentation

To enhance the generalization performance of our training data [22], we applied data augmentation using TrivialAugment [23], a parameter-free automatic augmentation method. We sampled the augmentation strength uniformly and repeated this process five times for each image. We combined these augmented versions with the original mel spectrograms to create six variations that were tensorized to form the training dataset. For the validation and testing datasets, tensorization was performed without any data augmentation.

To address class imbalance in the training data, we employed context-rich minority oversampling (CMO) [24]. The CMO integrates oversampling with CutMix[25], thereby improving the generalization performance of classifiers in imbalanced datasets. For CutMix, we selected the foreground image from an oversampled minority class dataset and the background image from the original dataset. We did not apply CMO to the validation or testing datasets. Considering the potential of larger batch sizes in the CMO to capture a broader range of features [24], we set the training batch size to 512 and maintained a batch size of 32 for the validation and testing data.

### Image classifier and ensemble learning

**Fig. 1:**
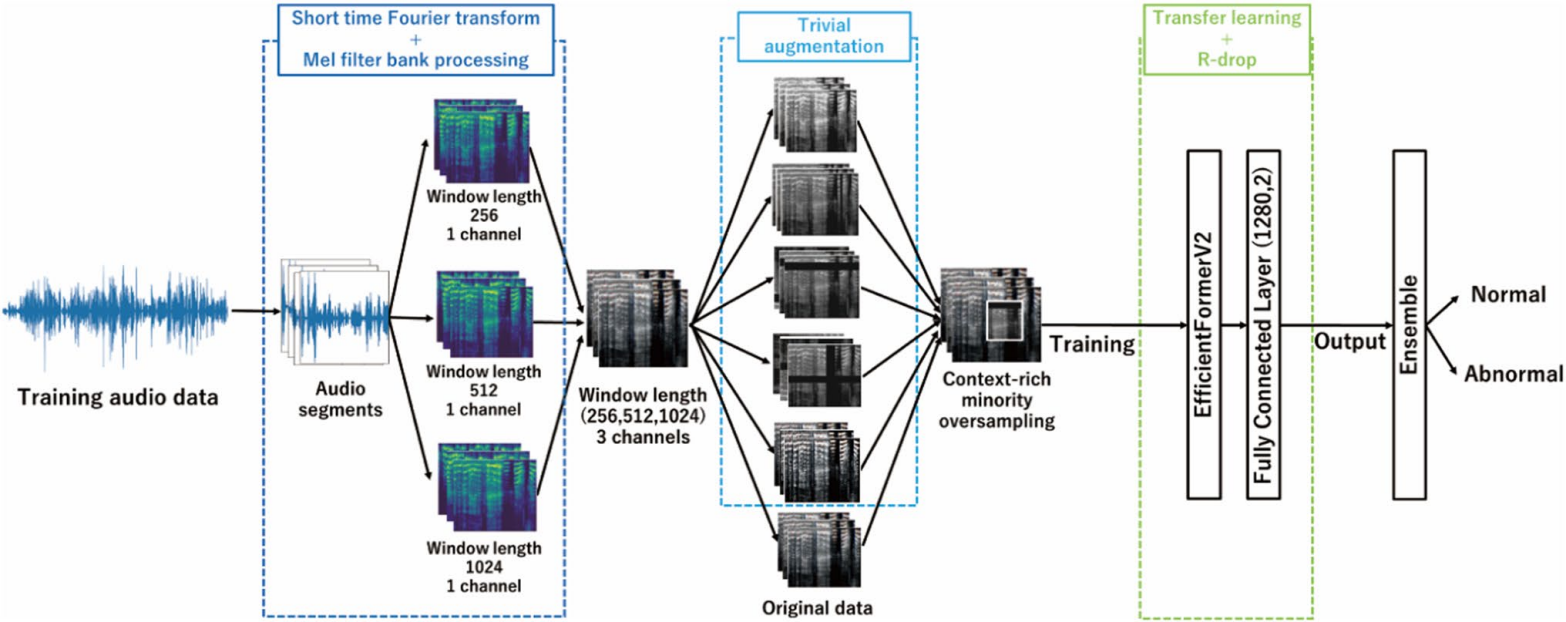
Learning flow on training data

Transfer learning [26] was used with the acquired training and validation data to train the classification model. We selected the EfficientFormer V2-L model [27] because of its balance between accuracy and computational efficiency, which is crucial for potential clinical applications. EfficientFormer is an advanced version of the Vision Transformer (ViT) designed to handle image data effectively while maintaining lower computational costs. While other architectures such as standard ViTs, convolutional neural networks (CNN), or multilayer perceptrons (MLPs) are available, EfficientFormer offers improved performance in terms of speed and resource utilization. The model was pre-trained on the ImageNet dataset [28], a large-scale database of annotated images widely used in computer vision research. We modified the model's fully connected layer to output two classes, indicative of the presence or absence of mental disorders, and froze the remaining layers to limit learning to the fully connected layer. Ensemble learning was employed to enhance the robustness and generalizability of the classification model. Ensemble methods combine predictions from multiple models to reduce variance and bias, leading to improved overall performance compared with individual models [29]. Specifically, we integrated the outputs from the models trained on different spectrogram representations, each capturing the unique time–frequency characteristics of the audio data. By aggregating these predictions, we aimed to increase the model's ability to recognize patterns associated with mental disorders in the voices of pregnant women. Figure 1 illustrates the overall learning flow of the training data. We conducted all analyses using Python version 3.8.16 (Python Software Foundation, Beaverton, OR, USA), PyTorch version 2.0.0, and CUDA version 11.8.

We did not employ cross-validation (CV) in this study for two primary reasons: First, implementing CV would have significantly increased the computational burden[30], making it impractical given our resource limitations and the lightweight nature of the model. Second, **t**he audio data were segmented into smaller, time-series-based segments. Applying CV in this context could inadvertently lead to data leakage, where segments from the same original audio recording appear in both the training and validation folds, compromising the integrity of the evaluation[31]. Instead of CV, we allocated distinct subsets for training, validation, and testing to ensure an independent evaluation of the model's performance.

### Outcome

We used G-mean as the primary outcome metric to evaluate mental disorders in pregnant women through voice data analysis. G-mean, defined as the geometric mean of sensitivity and specificity, provides a robust metric for imbalanced data. Our secondary outcomes included accuracy, sensitivity, specificity, precision, recall, F1 score, receiver operating characteristic area under the curve (ROC-AUC), and precision-recall area under the curve (PR-AUC). We performed DeLong's test [32] to assess the ROC-AUC results.

1 $${\text{G}} - {\text{mean }} = \sqrt {{\text{ Sensitivity}} \cdot {\text{Specificity }}} = \sqrt {\frac{TP}{{TP + FN}} \cdot \frac{TN}{{TN + FP}}}$$

2 $${\text{Accuracy }} = \frac{TP + TN}{{TP + TN + FP + FN}}$$

3 $$Precision = \frac{TP}{{TP + FP}}$$

4 $$Recall = \frac{TP}{{TP + FN}}$$

5 $$F_{1} score = 2 \cdot \frac{ Precision \cdot Recall }{{ Precision + Recall }} = \frac{ 2 \cdot TP}{{ 2 \cdot TP + FP + FN }}$$

6 $$True\; positive \;rate \;TPR = \frac{TP}{{TP + FN}}$$

7 $$False\; positive\; rate\; FPR = \frac{FP}{{FP + TN}}$$

TP: True positive; FP: False positive; TN: True negative; FN: False negative.

### Hyperparameter optimization

We optimized the hyperparameters using Optuna [33], a Bayesian optimization tool, and selected G-mean as our optimization metric. In each 20-epoch learning round, the best score was updated based on the G-mean of the validation data. If a learning round produced a score that surpassed the previous best score, we recorded the corresponding hyperparameters.

### Loss function and Optimizer

We evaluated both cross-entropy and focal loss functions [34]. For focal loss, we set the focusing parameter gamma (γ) between 2 and 4 and the alpha (α) parameter between 0.40 and 0.60. We selected the AdaBelief optimizer [35] by adjusting the learning rate between 1e and 4 and 1.0, and the β parameter from 0.880 to 0.990. The Adabelief follows a similar update rule to Adam while incorporating a mechanism that learns the confidence of the gradients. This allows it to adapt to the learning rate more effectively, thereby facilitating a faster convergence to the optimal solution and mitigating overfitting.

### Scheduler

We explored both the cosine learning rate scheduler [36] and the warm-up scheduler [37]. For the cosine scheduler, we adjusted the minimum learning rate from 1e–9 to 1e–5 and set the frequency of the learning rate changes per epoch between 1 and 3. For the warm-up scheduler, we similarly adjusted its parameters and set the number of warm-up repetitions to vary from one to three.

### Comparison

We selected the EPDS as our baseline comparator, as it is the most widely used screening tool in Japan [38], with substantial evidence supporting its effectiveness in pregnant women [39]. Considering findings from meta-analyses on the use of the EPDS to detect major depression during pregnancy [40], along with studies using the EPDS to screen for other psychiatric disorders [41], we established a cutoff point of 11 or higher.

## Results

**Fig. 2:**
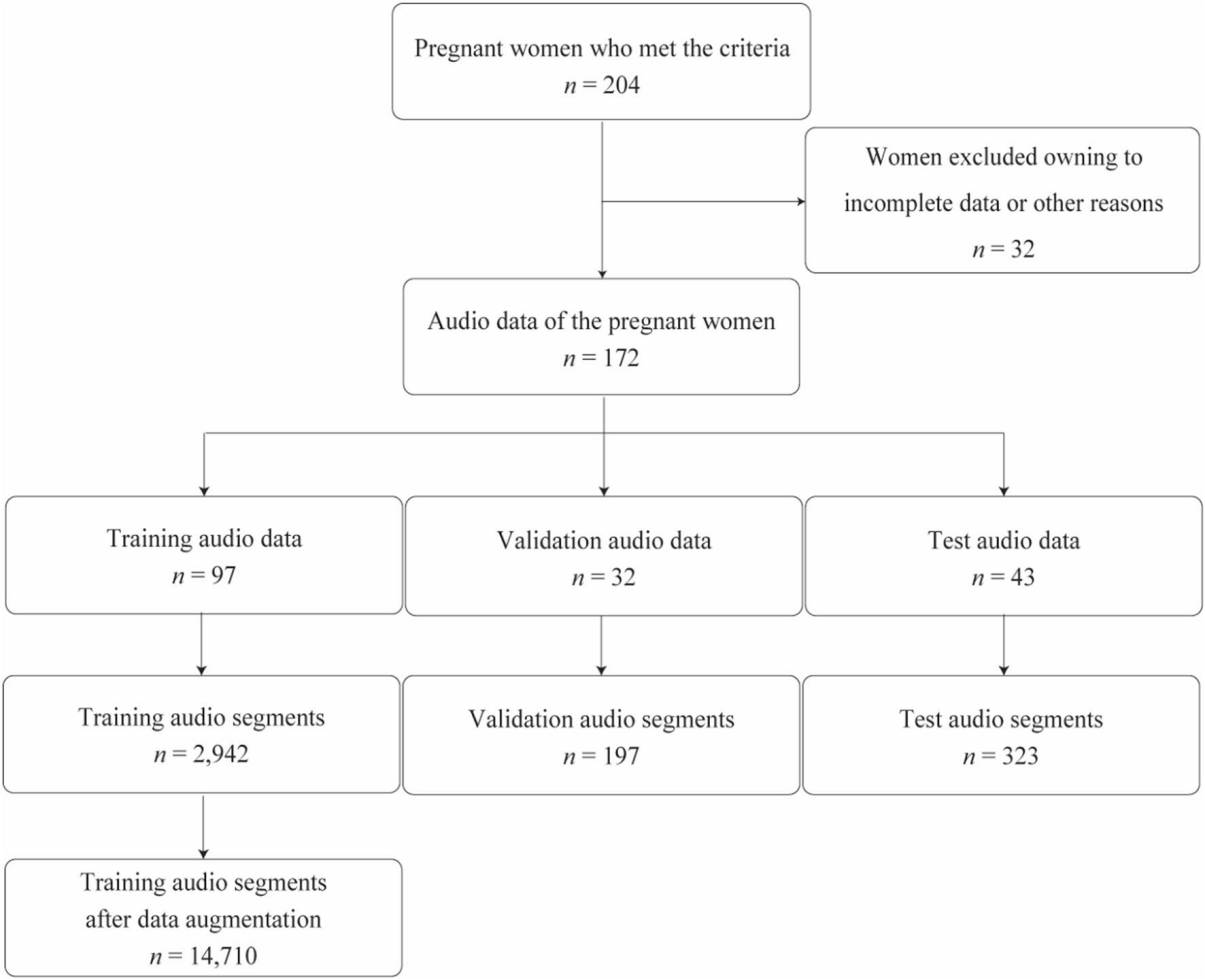
Processing flow

A total of 204 pregnant women who met the inclusion criteria were enrolled in this study. After excluding 32 participants due to insufficient voice recordings or incomplete medical records, 172 participants were included in the final analysis: 149 were classified as Class 0 (no mental disorder) and 23 as Class 1 (mental disorder present). Participants were allocated to training (n = 97, 56.4%), validation (n = 32, 18.6%), and test (n = 43, 25.0%) groups to ensure similar class distributions. Of the 23 participants in Class 1, 13 were assigned to the training set, 4 to the validation set, and 6 to the test set. The 4 Class 1 participants in the validation set had the following diagnoses: major depressive disorder (n = 1), bipolar disorder (n = 1), adjustment disorder (n = 1), and intellectual disability (n = 1). The 6 Class 1 participants in the test set were diagnosed with major depressive disorder (n = 2), bipolar disorder (n = 1), adjustment disorder (n = 1), anxiety disorder (n = 1), and intellectual disability (n = 1). All remaining Class 1 participants were included in the training set. Table 1 provides additional details regarding the overall distribution of psychiatric disorders among all study participants. The average audio duration across the dataset was 549.2 ± 356.0 s, with the training data averaging 556.0 ± 358.4 s, validation data 549.0 ± 350.2 s, and test data 549.2 ± 355.5 s. Segmenting the audio recordings resulted in 2,942 training segments (Class 0:2,503; Class 1:439), 197 validation segments (Class 0:163; Class 1:34), and 323 test segments (Class 0:297; Class 1:26). After data augmentation, the number of training segments increased to 14,710 (Class 0: 12,515; Class 1: 2,195). The class-wise data distribution is presented in Table 2. The performance metrics for the voice-based model and EPDS are summarized in Table 3, and the confusion matrices for both methods are presented in Table 4. In evaluating the voice model and EPDS, the voice model achieved a sensitivity of 1.00, whereas the EPDS demonstrated a specificity of 0.97. The EPDS also achieved an accuracy of 0.91 and a precision of 0.84. The voice model had a higher recall of 0.82 and a G-mean of 0.81. The F1 score was higher for the EPDS at 0.77. The ROC-AUC was 0.82 for the voice model and 0.74 for the EPDS, with no significant difference between the two methods (p = 0.759). Both tools had a PR-AUC value of 0.66 (Fig. 2).

**Table 1:** Participant Background

Baseline characteristics	Overall *n* = 172^1^	Mental disorders	
		Class 0 *n* = 149^1^	Class 1 *n* = 23^1^
*Type of Mental Disorder (%)*			
Major depressive disorder	10 (5.8%)	0 (0.0%)	10 (43.5%)
Schizophrenia	2 (1.2%)	0 (0.0%)	2 (8.7%)
Bipolar disorder	4 (2.3%)	0 (0.0%)	4 (17.4%)
Adjustment Disorder	5 (2.9%)	0 (0.0%)	5 (21.7%)
Autism spectrum disorder	1 (0.6%)	0 (0.0%)	1 (4.3%)
Sleep disorder	1 (0.6%)	0 (0.0%)	1 (4.3%)
Eating disorder	2 (1.2%)	0 (0.0%)	2 (8.7%)
Anxiety disorder	2 (1.2%)	0 (0.0%)	2 (8.7%)
Intellectual disability	4 (2.4%)	0 (0.0%)	4 (17.4%)
Alcohol dependence	1 (0.6%)	0 (0.0%)	1 (4.3%)
*Total EPDS score*	4 ± 4	4 ± 4	8 ± 6
*Total EPDS score > 10*	17 (9.9%)	9 (6.0%)	8 (34.8%)

^1^Number (Percentage); n (%); Mean ± SD; n

**Table 2:** Class-wise Data Distribution Before and After Augmentation

Dataset	Class	Before Augmentation	After Augmentation
Training	Class 0	2,503	12,515
	Class 1	439	2,195
Validation	Class 0	163	163
	Class 1	34	34
Test	Class 0	297	297
	Class 1	26	26

**Table 3:** Confusion Matrix of Voice and EPDS for Test Data. EPDS: Edinburgh postnatal depression scale

		True label			
		Voice		EPDS	
		Positive	Negative	Positive	Negative
Predicted label	Positive	6	13	3	1
	Negative	0	24	3	36

**Table 4:** Evaluation Metrics of Voice and EPDS for Test Data

	Sensitivity	Specificity	G-mean	Accuracy	Precision	Recall	F1 score	ROC-AUC	PR-AUC
Voice	1.00	0.65	0.81	0.70	0.66	0.82	0.63	0.82	0.66
EPDS	0.50	0.97	0.70	0.91	0.84	0.74	0.77	0.74	0.66

EPDS: Edinburgh postnatal depression scale; G-mean: geometric mean; ROC-AUC: receiver operating characteristic area under the curve; PR-AUC: precision-recall area under the curve

## Discussion

In this study, we developed a machine learning model that analyzes the voices of pregnant women to screen for mental disorders. Our results indicate that voice analysis can serve as an objective indicator for diagnosing mental illness in this population, achieving a sensitivity of 1.00 and a G-mean of 0.81. These findings suggest that our voice-based approach has potential utility in perinatal mental health screening, comparable to conventional methods, such as EPDS.

Previous studies have explored the use of vocal characteristics to detect mental health conditions. A previous study [8] examined acoustic features associated with depression, including reduced pitch range and decreased speech rate. Another study [9] developed a deep learning model capable of detecting depression from speech data. However, these studies primarily focused on the general population or specific disorders, such as depression, with limited attention to pregnant women and multiple mental disorders. Hormonal changes during pregnancy are known to affect vocal characteristics [10], suggesting that voice analysis could be particularly relevant in this population. Our study addresses this gap by focusing on pregnant women and including various mental disorders, not just depression. In tasks involving emotion classification using voice, melspectrograms have shown better performance than the mel-frequency cepstrum coefficient [42], likely owing to their better resistance to high-frequency noise. Melspectrograms also effectively reduce dimensionality while preserving essential voice-signal information with minimal loss of information [43]. Furthermore, it has been shown that it is possible to use transition learning with spectrograms, even with pre-trained models on non-speech image sets [20]. Given the challenge of collecting extensive voice data from pregnant women with mental disorders, fine-tuning pre-trained models is an efficient approach for maintaining accuracy with limited data. To address the data imbalance, we implemented the CMO. This sampling strategy ensures equality between the majority and minority classes in each mini-batch, reducing bias. By learning independently from these mini-batches, the model achieved a bagging-like effect, thereby decreasing the overall prediction variance [44]. The voice-based approach demonstrated higher sensitivity, likely due to the imbalance correction favoring the minor class, while the EPDS showed higher specificity, aligning with previous studies [11].

This study had several limitations. First, the relatively small sample size and imbalance between Class 0 and Class 1, especially after data augmentation, may limit the generalizability of our findings. Although we used the CMO to mitigate this imbalance, it may have introduced artificial patterns and biased the model. Future studies should collect larger and more balanced datasets to enhance the robustness of the model. Second, we did not perform cross-validation owing to the computational constraints associated with our lightweight EfficientFormer model and the potential for data leakage arising from the segmentation of time-series audio data. Implementing cross-validation would have significantly increased computational costs and could have compromised the independence of the training and validation sets. Third, although only specific models were used in this study for efficiency and accuracy, models using CNNs, ViTs, and decision trees should be evaluated. Fourth, although we used the EPDS for comparison, it is primarily designed to screen for postpartum depression and may not capture all perinatal mental disorders. Fifth, our method may have detected vocal disorders rather than mental disorders. However, since our labels were based on confirmed mental health diagnoses and vocal disorders are rare in this population [13], this concern is likely minimal. Finally, the use of noise-reduction techniques may have led to some loss of information. Recording in a noise-free environment is ideal but may not be feasible in clinical settings.

## Conclusion

We developed a lightweight machine learning model to analyze pregnant women's voices for screening various mental disorders, achieving high sensitivity and demonstrating the potential of voice analysis as an effective and objective tool in perinatal mental health care. This approach overcomes the limitations of traditional self-reported questionnaires and offers a promising tool for early detection and intervention in perinatal mental health.

## Data Availability

The datasets produced and/or examined during the present investigation were obtained from the relevant author following a reasonable inquiry.
